# Probability Method of Molecular Size, Polydispersity, and Branch Unit Distribution Function for the Three-Dimensional Polymers: A Continuation of Flory’s Work in 1941

**DOI:** 10.1021/acs.macromol.5c00090

**Published:** 2025-05-21

**Authors:** Yinghao Li, Jing Lyu, Wenxin Wang

**Affiliations:** Charles Institute of Dermatology, School of Medicine, 8797 University College Dublin, Dublin 4 D04 V1W8, Ireland

## Abstract

This study extends Flory’s 1941 foundational work on three-dimensional step-growth polymerization (SGP) with trifunctional branching units. Flory laid the groundwork for understanding polymer size distribution back then, however, he did not fully explore the polydispersity and/or average degree of polymerization in A_2_ + A_3_ and A_2_ + A_3_ + B_2_ types of systems. As the demand for advanced three-dimensional polymer materials grows, a more comprehensive theoretical framework is required. In this work, new analytical expressions are derived for the number-average (*X*_n_), weight-average (*X*_w_) degree of polymerization, and polydispersity (Đ) of trifunctional branched polymers through Flory’s probability method. Additionally, we introduce the concept of Branch Unit Distribution (BUD), which has not been previously investigated. This new theoretical concept is validated through comparison with both experimental results and Monte Carlo simulations. Our findings provide new insights into the internal structure of three-dimensional branched polymers, offering valuable guidance for future design and development of novel materials from SGPs.

## Graphical abstract

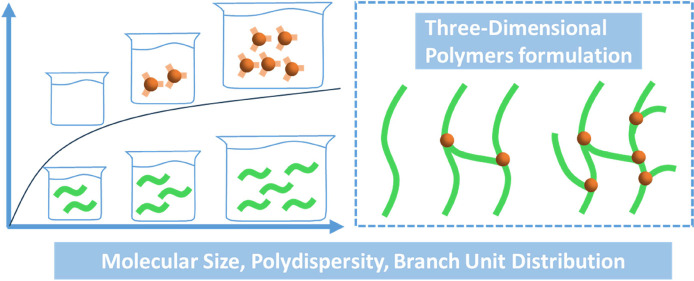

## Introduction

In the 20th century, Flory made significant contributions to the development of the fundamental theory of step-growth polymerization (SGP).^1^ His work has been a cornerstone of SGP research and continues to be widely used today. However, his study on three-dimensional polymers from SGPs is still far from fully understanding the internal structure and composition.^2,3^ Flory’s studies on molecular size distribution in three-dimensional polymers were published in *Journal of the American Chemical Society* from 1941 to 1952: Molecular Size Distribution in Three Dimensional Polymers. I to VI),^4–9^ including the investigation of the composition of branched polymer components, gelation behavior, and the average molecular weight of some typical polymerization systems (AB_f_).^9^ Since Flory’s pioneering contributions, polymerization theory has advanced significantly. This included progress in theoretical research methods, evolving from Flory’s initial probability approach to more sophisticated techniques^10^ such as statistical mechanics,^11–15^ generating functions,^16,17^ and kinetic approaches.^18–21^ The scope also extended to various homopolymer and copolymer systems, such as A_f_B_g_, AB_2_ + AB,^18^ AB_g_ + B_f_,^19,20^ and AB_g_ + AB_h_ + B_f_,^21^ etc.

Despite these advancements, certain polymerization systems remain unexplored—particularly those synthesized via the A_2_ + A_n_ + B_2_ method (a commonly used method to synthesize SGP polymers).^22–24^ This approach involves introducing a small amount of multifunctional monomers into a linear system to create branched structures. A prominent example is poly­(β-amino ester) (PAE), an important polymer system with significant applications in gene and drug delivery.^25–27^ PAE is typically synthesized through Michael addition, combining a diacrylate, an amine, and a multifunctional acrylate or amine monomer. As research progressed, scientists have increasingly recognized the critical connection between the precise structure of polymers and their performance and properties in applications.

In order to guide the good structural control of polymers, it is necessary to have underlying theoretical guidance for polymer synthesis. Furthermore, our recent studies have highlighted the importance of a new structural factor–Branch Unit Distribution (BUD) in three-dimensional branched polymer materials, which can significantly affect their gene delivery performance.^28^ It is worth noting that this BUD property differs from the traditional branching degree (BD),^29^ as it refers to the distribution of branched units across polymer fractions separated by molecular weight. By fractionating the polymer according to molecular weight and characterizing these fractions individually, it is observed that the ratio of branched monomers to linear monomers (BUD) varies among these molecular weight-separated fractions. In the lower molecular weight fractions, the content of branched monomers is relatively low. As the molecular weight increases, the proportion of branched monomers gradually rises and eventually stabilizes. However, the analytical expression of BUD has never been studied and reported.

In this study, we aimed to extend Flory’s work by establishing analytical expressions for *X*_w_ (weight-average degree of polymerization), *X*_n_ (number-average degree of polymerization), and *Đ* (polydispersity) of polymerization through Flory’s probability method with A_2_ + A_3_ and A_2_ + A_3_ + B_2_ types of monomers. The A_2_ + A_3_ system contains bifunctional A_2_ monomers and trifunctional A_3_ monomers. The two A groups are capable of reacting with each other. Some typical examples include: the reaction of silanediol and silanetriol monomers to form polysiloxane, the reaction of ethylene glycol and triol to produce PEG, the reaction of dicarboxylic acid and tricarboxylic acid monomers to form poly­(anhydride), the reaction of diamine and triamine monomers to form polyurea, and the reaction of diisocyanate and triisocyanate monomers to form polycyanurate. The A_2_ + A_3_ + B_2_ system consists of bifunctional A_2_ monomers, trifunctional A_3_ monomers, and bifunctional B_2_ monomers. The A and B groups can react with each other. This type of system is more common, such as in the reactions of carboxylic acids with alcohols, acrylates with amino monomers, and bisphenol A-based phenolic resins used in polyurethane production, among others. Moreover, the predicted results from the newly developed theoretical framework and derived equations were validated by being compared with those from experiments and Monte Carlo (MC) simulations. Furthermore, on top of *X*_n_, *X*_w_, and *Đ*, the Flory framework of SGP was also extended to derive the analytical formula of BUD in the A_2_ + A_3_ and A_2_ + A_3_ + B_2_ polymerization systems, providing theoretical guidance in the new chemical characteristics of these branched materials.

## Results and Discussion

### Theoretical Treatment

This study builds upon Flory’s 1941 work, maintaining consistency with its theoretical framework. The two main assumptions remain: (1) all functional groups exhibit equal reactivity regardless of their position, and (2) intramolecular reactions are absent. Moreover, being the same as the reaction systems studied by Flory, this work is also focused on the equimolar SGP systems with trifunctional branching units: (i) A_2_ + A_3_; (ii) A_2_ + A_3_ + B_2_. The polymerization process can be represented by Scheme 1, A and B representing two types of functional groups, A_2_ and B_2_ are difunctional monomers, and A_3_ is a trifunctional molecule. Flory’s original framework^5^ assumed that the trifunctional branching unit (A_3_) was massless to simplify derivations. While this assumption did not impact the species distribution derivation, it restricted the analysis of properties directly associated with branching units, such as BUD. Therefore, to make the theoretical treatment more realistic and to enable the derivation of the expression of BUD, in this work, the mass (size) of the A_3_ branching unit was included, which was treated to be the same as that of the linear monomers A_2_ and B_2_. Apart from this, all other assumptions are consistent with the classic Flory framework, including the equal reactivity of all A functional groups in the A_3_ and A_2_ monomers, and the inability of intramolecular reactions to occur.

**Scheme 1:**
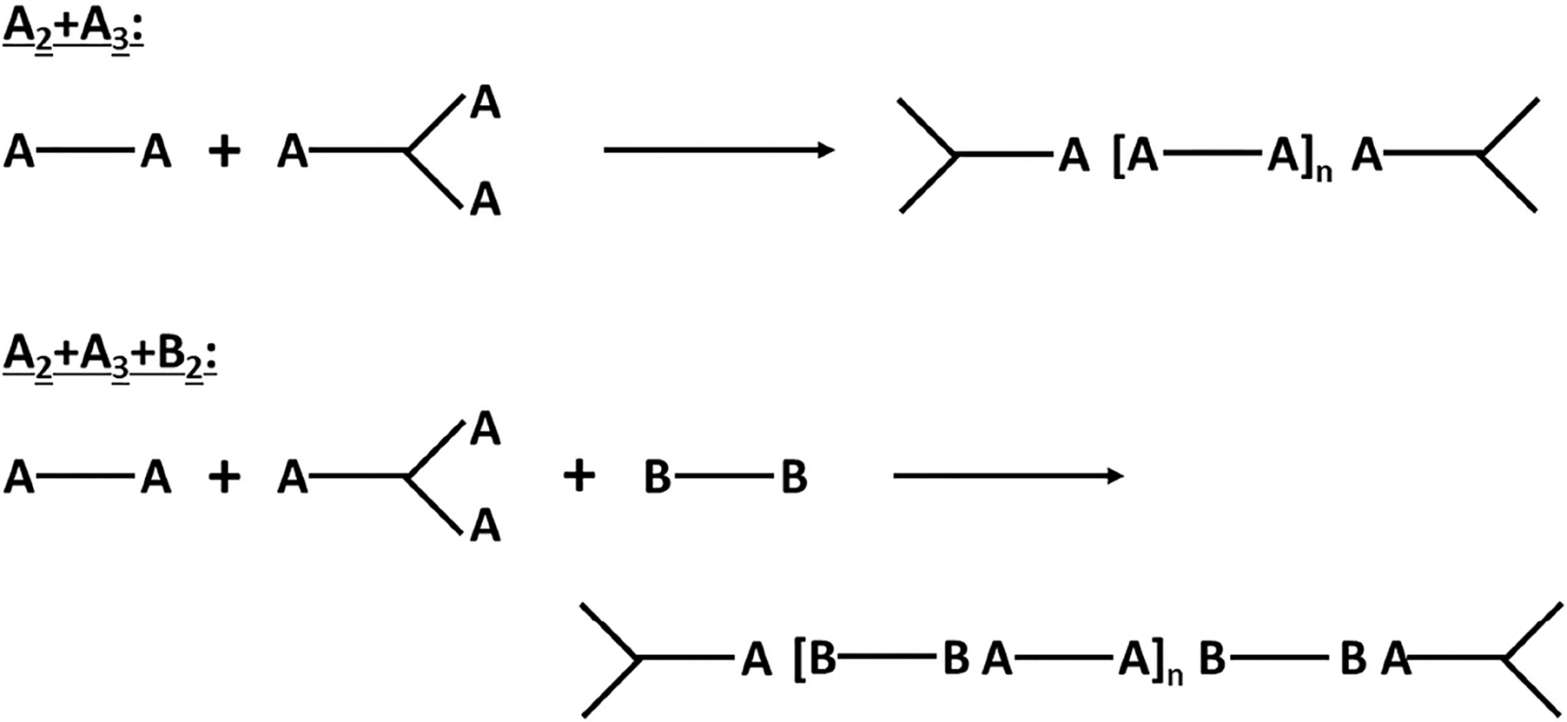
Schematic Representation of the Polymerization Process in Equimolar Step-Growth Polymerization with Trifunctional Branching Units

The symbols used in the following derivation and their physical meanings are the same as Flory’s original definitions,^4,5^ where ρ (eq 1) is the ratio of A group belonging to branch units to the total number of A group.

$$\rho =\frac{3A_{3}}{2A_{2}+3A_{3}}$$ 1

α (eq 2 in A_2_ + A_3_ system, and eq 3 in A_2_ + A_3_ + B_2_ system) is the critical branching probability, which refers to the probability that any given one of the functional groups of a branch unit leads, via a sequence of bifunctional units, to another branch rather than to a terminal group.

$$\alpha_{I}=\frac{P_{A}\rho }{1-P_{A}(1-\rho )}$$ 2

$$\alpha_{\mathrm{II}}=\frac{{P_{A}}^{2}\rho }{1-{P_{A}}^{2}(1-\rho )}$$ 3

where *P*_A_ is the reaction extent of A.

In subsequent calculations, *z* represents the number of chains in a molecule, and *n* represents the number of trifunctional branching units A_3_ in a molecule, and they follow the following relationship: *z* = 2*n* + 1.

### A_2_ + A_3_ System—The Average Degree of Polymerization and Polydispersity

The relatively simple A_2_ + A_3_ system was first considered. To derive the expression of number-average, weight-average degree of polymerization and polydispersity, the first step is to establish the recursive relationship for *W*_*n*_ – the probability that a randomly selected chain from the polymer mass is part of a network composed of *z* chains combined by *n* trifunctional branch units, which is defined in eq 4 as Flory’s work in 1941 (letting β = α­(1 – α)).^5^ *W*_*n*_ is also the weight fraction of networks possessing *n* branching units. Therefore, the weight fraction *W*_*z*_ of networks composed of *z* chains (where *z* = 2*n* + 1) is described as eq 5.

$$W_{n}=(1-\alpha {)}^{2}\frac{(2n+2)!\beta^{n}}{(n+1)!(n+2)!}$$ 4

$$W_{z}=\frac{(1-\alpha {)}^{2}(z+1)!\beta^{(z-1)/2}}{\left[\frac{z+1}{2}\right]!\left[\frac{z+3}{2}\right]!}$$ 5

The next step is to establish *Q* *xz* – the probability that there are *x* units (including the branching units A_3_) in a network of *z* chains selected at random. The same as Flory’s work, *Q*_*xz*_ can be defined as eq 6, where *y* refers to a chain selected at random containing *y* bifunctional units, and *q* is the probability of continuation of a chain, i.e., the mean probability that a given functional group has condensed with a functional group of a bifunctional unit (eq 7).

$$Q_{\mathrm{xz}}=\sum [(1-q)q^{y_{1}-1}(1-q)q^{y_{2}-1}\cdots (1-q)q^{y_{z}-1}]$$ 6

$$q=P_{A}(1-\rho )$$ 7

Due to the consideration of the mass/size of the branching unit A_3_ in this work, the summation over *y*_1_ to *y*_*z*_ should be rewritten as below, which equals the total number of units in a network selected at random (*x*) excluding the number of trifunctional units *n*:

$$y_{1}+y_{2}+\cdots y_{z}=x-n$$ 8

Combining eq 6 and eq 8, *Q*_*xz*_ can be revised to a new expression with the trifunctional branching units taken into account – *Q*′*_*xz*_*, which represent the mean probability that a given functional group (include bifunctional and trifunctional units) has condensed with a functional group of a bifunctional unit:

$$Q_{xz}^{\prime }=q^{(x-n)-z}(1-q{)}^{z}\frac{((x-n)-1)!}{(z-1)!((x-n)-z)!}$$ 9

Based on this, of the total quantity of *z*-chain species, the weight fraction composed of *x* units with (*x* – *n*) A_2_ and *n* A_3_ is xQxz′(z(1−q))+n , where l/(l – *q*) is the average number of bifunctional units per chain, and (*z*/(l – *q*)) + *n* is the average number of units in a *z*-chain network. Of the total polymer, the weight fraction composed of *x*-unit, *z*-chain species is given by WzxQxz′(z(1−q))+n . Therefore, the weight fraction of *x*-unit species, regardless of the number of chains can be derived as eq 10, meaning the ratio of the total amount of A_2_ and A_3_ units in the molecules containing *x* units to the total amount of A_2_ and A_3_ in the system.

wx′=∑z=1,3,5⋯∞WzxQxz′(z(1−q))+n 10

To validate that the introduction of the mass/size of A_3_ does not affect the prediction of the distribution of species in three-dimensional systems with trifunctional branching units, the newly derived *w*′*_*x*_* was compared with Flory’s *w*_*x*_ (eq 11)^5^ in an A_2_ + A_3_ system with A_2_:A_3_ = 1:0.1 and *P*_A_ = 0.92. Moreover, the numerical method – MC simulation was also employed to simulate the A_2_ + A_3_ reaction under the same conditions for comparison (see detailed simulation description in Supporting Information, Scheme S1).

$$w_{x}=\sum_{z=1,3,5\cdots }^{\infty }W_{z}Q_{xz}x(1-q)/z$$ 11

As shown in Figure 1A,B, the newly derived *w*′*_*x*_* equation produced weight fraction values of species in the system comparable to those predicted by the Flory *w*_*x*_ equation. Moreover, the weight fraction evolution trends under different reaction extents (*P*_A_) were similar under both equations – the weight fraction of oligomers significantly decreased with the increase of *P*_A_, and the species with a high degree of polymerization became more and more. These findings confirm that within the Flory framework, accounting for the mass/size of the branching unit does not significantly impact the predicted weight fraction distribution in three-dimensional polymer systems with trifunctional branching units. Moreover, the results from the MC simulations (Figure 1C) are nearly identical to the theoretical predictions, which verified the accuracy of the theoretical derivation.

**Figure 1:**
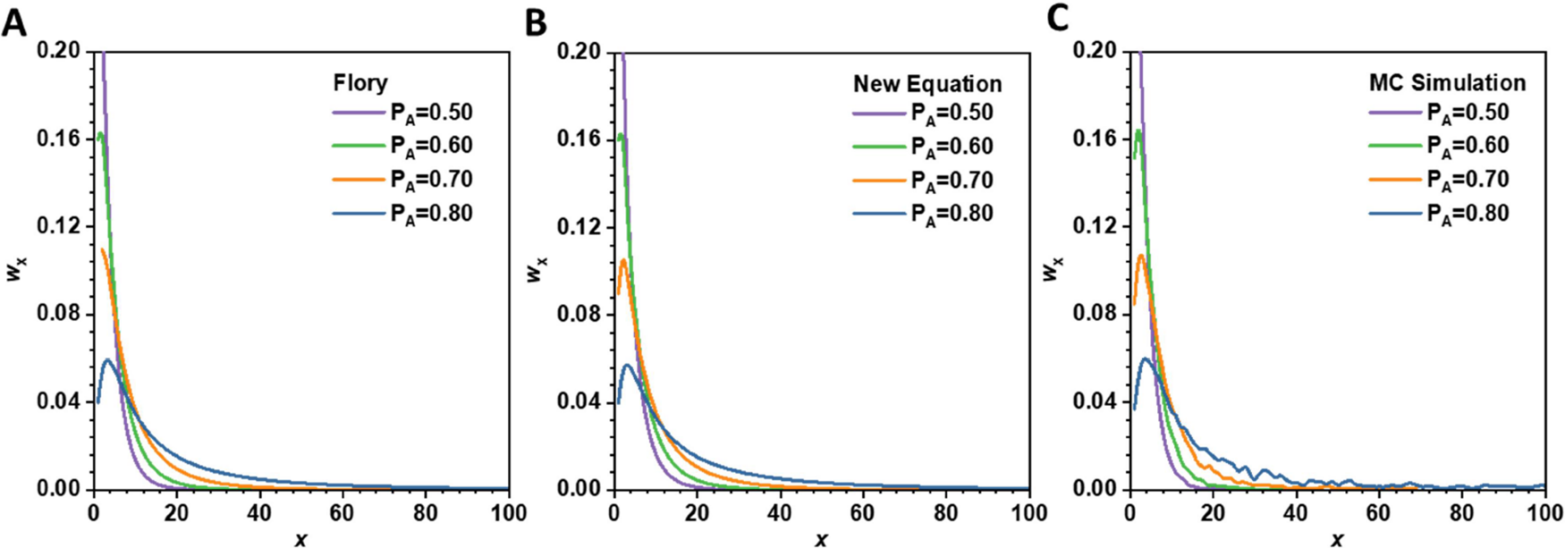
Weight fraction distributions of species in A_2_ + A_3_ system based on three different methods. (A) Flory’s equation *w*_*x*_ (eq 11), (B) New equation *w*′*_*x*_* (eq 10), and (C) Monte Carlo (MC) simulation *w*_*x*_. The weight fraction distributions are plotted against *x* for *P*_A_ = 0.80, 0.70, 0.60, and 0.50, respectively. The initial molar ratio of A_2_:A_3_ was set as 1:0.1. The integrated area under each curve extrapolated to infinity is unity.

After proving the reliability of the amended weight fraction distribution formula – *w*′*_*x*_*, the number-average, weight-average degree of polymerization and polydispersity for the A_2_ + A_3_ system were developed in the next step. First, since *w*′*_*x*_* represents the mass distribution of polymer components, the weight-average degree of polymerization of three-dimensional polymers with trifunctional branching units is

X®w=∑xwx′=∑(x∑z=1,3,5⋯∞WzxQxz′(z(1−q))+n) 12

By considering the physical definition of the weight fraction of *x*-mer (polymeric molecules composed of *x* units), *w*′*_*x*_* can also be written as

$$w_{x}^{\prime }=\frac{xN_{x}}{N_{0}}$$ 13

where *N*_*x*_ is the number of *x*-mers, *N*_0_ is the initial total number of monomer units. Then, the number-average degree of polymerization can be obtained:

$${\overset{®}{X}}_{n}=\frac{\sum xN_{x}}{\sum N_{x}}=\frac{N_{0}\sum w_{x}^{\prime }}{\sum N_{x}}=\frac{N_{0}\sum w_{x}^{\prime }}{N_{0}\sum \frac{w_{x}^{\prime }}{x}}=\frac{\sum w_{x}^{\prime }}{\sum \frac{w_{x}^{\prime }}{x}}$$ 14

Based on [Disp-formula eq12] and 14, the polydispersity *Đ* can be calculated as

$$Đ=\frac{{\overset{®}{X}}_{w}}{{\overset{®}{X}}_{n}}$$ 15

Therefore, to evaluate the capability of the newly derived equations ${\overset{®}{X}}_{w}$ , ${\overset{®}{X}}_{n}$ , and *Đ*, their prediction results for the A_2_ + A_3_ system (with A_2_:A_3_ of 1:0.1) were compared with the results from corresponding MC simulations. As shown in Figure 2, the results from the MC simulations for the ideal system are almost identical to the theoretical predictions.

**Figure 2:**
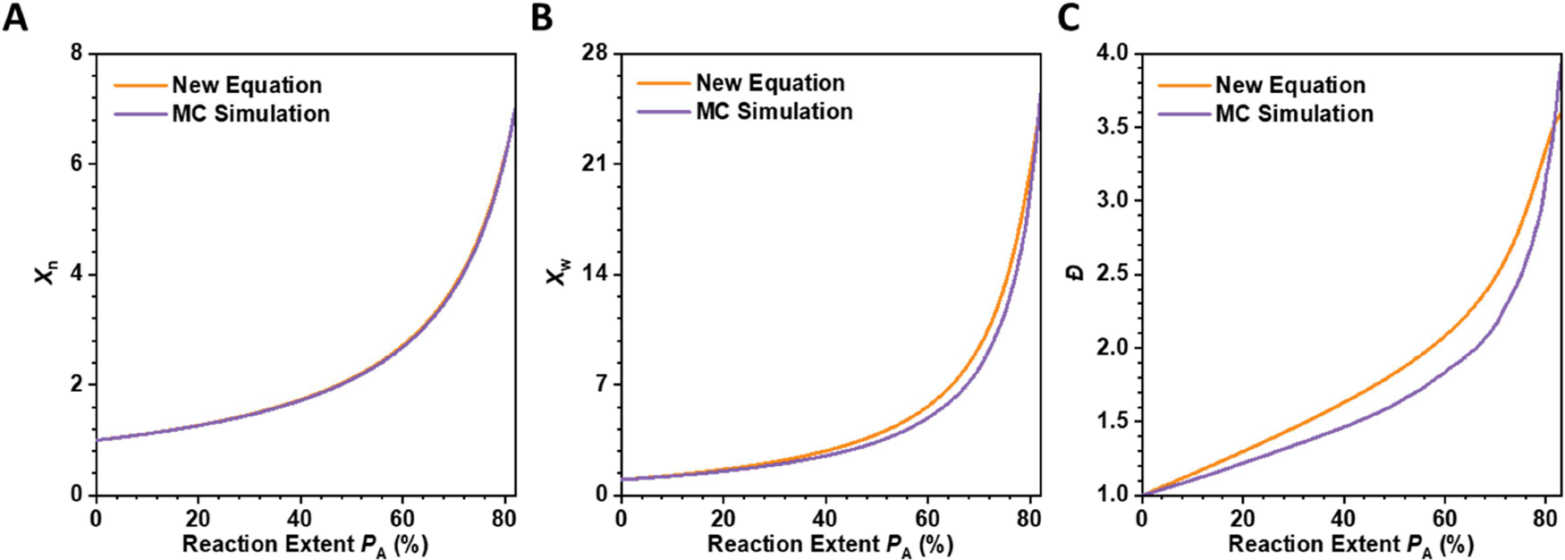
Polymerization behavior of A_2_ + A_3_ system. Evolution of the (A) weight-average degree of polymerization (*X*_w_), (B) number-average degree of polymerization (*X*_n_), and (C) polydispersity (Đ) according to the new equations proposed in this study (eqs 12, 14, and 15), and Monte Carlo simulation. The initial molar ratio of A_2_:A_3_ was set as 1:0.1.

### A_2_ + A_3_ System—Branch Unit Distribution (BUD)

After completing the derivation of molecular size and polydispersity, we further explore the internal structure of polymers. Our recent experiments^28^ have demonstrated the phenomenon of different BUD for copolymer systems containing multiple different functional monomers (containing both A_2_ and A_3_). This phenomenon refers to the difference in the ratio of A_2_ and A_3_ monomers in polymer components of different sizes, which can greatly affect the properties of polymer materials. However, no one has ever noticed this phenomenon before, and theoretical research on this type of distribution is still blank. This section aims to further derive the expression of BUD (i.e., the ratio of branching unit A_3_ to linear unit A_2_ in polymer components of different sizes within the molecule) of A_2_ + A_3_ system based on the *w*′*_*x*_* derived in the above section.

Based on *w*′*_*x*_*, the proportion of branching unit A_3_ in *x*-unit molecules to the total mass (*wb*_*x*_), which refers to the ratio of the total amount of A_3_ units in the molecules containing *x* units to the total amount of A_2_ and A_3_ in the system, was calculated as

wbx=nxwx′=∑z=1,3,5⋯∞nxWzxQxz′(z(1−q))+n 16

Similarly, the proportion of linear unit A_2_ (the ratio of the total amount of A_2_ units in the molecules containing *x* units to the total amount of A_2_ and A_3_ in the system) can be obtained in eq 17.

wlx=∑z=1,3,5⋯∞x−nxWzxQxz′(z(1−q))+n 17

Combining eqs 16 and 17, BUD, i.e., the proportion of branched unit A_3_ to linear unit A_2_ in a molecule of *x* units can be obtained as below:

$${\mathrm{BUD}}_{x}=\frac{Wb_{x}}{Wl_{x}}$$ 18

However, in real experiments, it is impossible to truly separate and characterize each individual molecule. In the methods used for separation, whether through stepwise precipitation or column chromatography, what is obtained is a series of polymer components with similar molecular weights. Therefore, for the analysis of BUD, a more meaningful statistic is to group together molecules of similar sizes (from *x* units to *x* + *gap* – 1 units). *gap* represents the unit size of each group (an example is shown in Scheme S4). At this point, the weight-average degree of polymerization of this series of molecules is denoted as $\overset{®}{x}$ .

x®x=∑xx+gap−1xwx′∑xx+gap−1wx′=∑xx+gap−1(x∑z=1,3,5⋯∞WzxQxz′(z(1−q))+n)∑xx+gap−1(∑z=1,3,5⋯∞WzxQxz′(z(1−q))+n) 19

By combining these molecules, the *w*′*_*x*_* can be used to calculate the average ratio of A_3_ monomers to A_2_ monomers in the polymer component with a weight-average degree of polymerization $\overset{®}{x}$ . Consequently, this leads to a series of discrete data points:

$${\mathrm{BUD}}_{\overset{®}{x}}=\frac{\sum_{x}^{x+gap-1}w_{x}\frac{Wb_{x}}{Wl_{x}}}{\sum_{x}^{x+gap-1}w_{x}},\;x=1,gap+1,2gap+1,3gap+1,\cdot \cdot \cdot$$ 20

Based on eqs 19 and 20, the BUD curve can be generated (Figure 3A–C and Scheme S5) under different reaction extents. MC simulations (see detailed simulation description in Supporting Information, Scheme S2) were also performed for comparison (Figure 3D–F). Overall, despite the absence of some MC data in the oligomer region (where molecular sizes are below 3), the formula and MC simulations displayed consistent evolutionary trends and yielded the same values of BUD. This further reinforces the reliability of both the new theoretical model and the simulation results. Moreover, the choice of the *gap* value does not affect the overall trend of the BUD and its corresponding A_3_/ A_2_ ratio. This means that we can determine the BUD of the entire polymer based on the weight-average molecular weight of the separated components and the average ratio of A_3_ monomers to A_2_ monomers.

**Figure 3:**
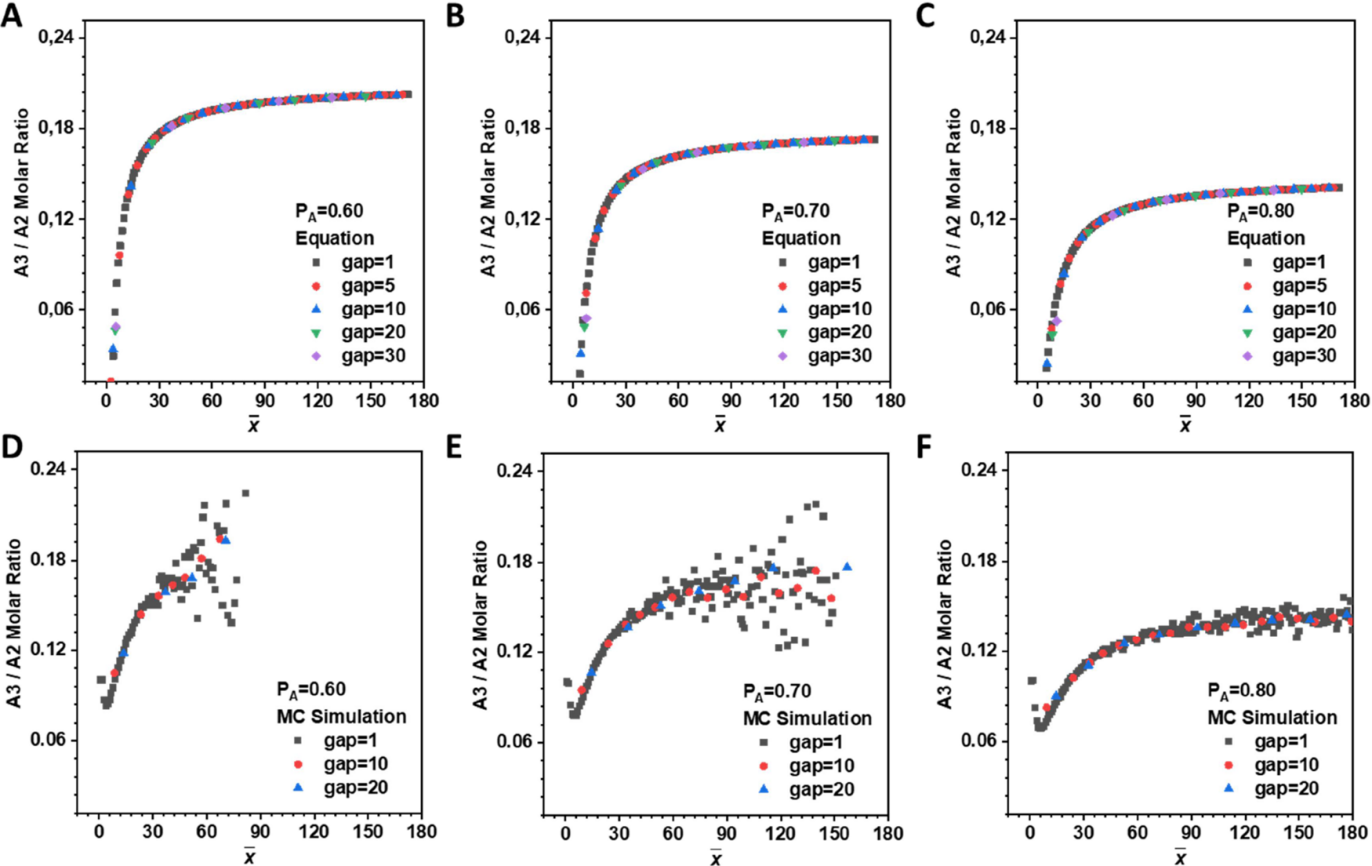
BUD (molar ratio of A_3_ to A_2_ in A_2_+ A_3_ system, obtained from eqs 19 and 20) at (A) *P*_A_ = 0.6, (B) *P*_A_ = 0.7, (C) *P*_A_ = 0.8, and from the MC simulation at (D) *P*_A_ = 0.6, (E) *P*_A_ = 0.7, and (F) *P*_A_ = 0.8. The initial molar ratio of A_2_: A_3_ was set as 1:0.1.

### A_2_ + A_3_ + B_2_ System—The Average Degree of Polymerization and Polydispersity

With the above insights, a more common experimental system – A_2_ + A_3_ + B_2_ was studied following a similar theoretical derivation process.

In the A_2_ + A_3_ + B_2_ system, the ratio of the number of A and B monomers is defined as γ (eq 21), where *r* is the ratio of the number of A and B functional groups (eq 22, set as 1 here to represent the equimolar system). Following the definition in the above A_2_ + A_3_ system, *x* is the number of A_2_ and A_3_ units in a network of *z* chains selected at random, therefore, the number of B_2_ units in a network can be described as *b* (eq 23).

$$\gamma =\frac{A_{2}+A_{3}}{B_{2}}=\frac{(3-\rho )r}{3}$$ 21

$$r=\frac{2A_{2}+3A_{3}}{2B_{2}}$$ 22

$$b=\frac{x}{\gamma }$$ 23

Based on this, of the total quantity of *z*-chain species, the weight fraction composed of (*x* + *b*) units ((*x* – *n*) A_2_, *n* A_3_, and *b* B_2_) can be obtained as (x+b)Qxz′/WAB(z(1−q))+n+b , where *W*_AB_ (eq 24) is the normalized mass, which is used to calculate the total mass fraction after introducing B_2_ units.

$$W_{\mathrm{AB}}=1+1/\gamma$$ 24

Then, the weight fraction of (*x* + *b*) unit species (the ratio of the total amount of A_2_, A_3_, and B_2_ units in the molecules containing (*x* + *b*) units to the total amount of A_2_, A_3_, and B_2_ in the system), regardless of the number of chains–*w*′_*x*(AB)_ is expressed as eq 25 (Figure S1).

wx(AB)′=∑z=1,3,5⋯∞Wz(x+b)Qxz′/WAB(z(1−q))+n+b 25

Then, by referring to eqs 21–25, ${\overset{®}{X}}_{n}$ , ${\overset{®}{X}}_{w}$ and *Đ* for the A_2_ + A_3_ + B_2_ system can be obtained (eqs 26–28). The curves of ${\overset{®}{X}}_{n}-P_{A}$ , ${\overset{®}{X}}_{w}-P_{A}$ and *Đ* – *P*_A_ are shown in Figure S2.

X̅w=∑(x+b)wx(AB)′=∑((x+b)∑z=1,3,5⋯∞Wz(x+b)Qxz′/WAB(z(1−q))+n+b) 26

$${\overset{®}{X}}_{n}=\frac{\sum (x+b)N_{x}}{\sum N_{x}}=\frac{N_{0}\sum w_{x(\mathrm{AB})}^{\prime }}{\sum N_{x}}=\frac{N_{0}\sum w_{x(\mathrm{AB})}^{\prime }}{N_{0}\sum \frac{w_{x(\mathrm{AB})}^{\prime }}{x+b}}=\frac{\sum w_{x(\mathrm{AB})}^{\prime }}{\sum \frac{w\prime_{x(\mathrm{AB})}}{x+b}}$$ 27

$$Đ=\frac{{\overset{®}{X}}_{w}}{{\overset{®}{X}}_{n}}$$ 28

Given the prevalence of the A_2_ + A_3_ + B_2_ system in experiments, the newly derived formulas were directly validated against experimental data. A typical A_2_ + A_3_ + B_2_ reaction was conducted, with 1,4-butanediol diacrylate (BDA) selected as the A_2_ monomer, trimethylolpropane triacrylate (TMPTA) as the A_3_ monomer, and N,N′-dimethyl-1,6-hexanediamine (DHD) as the B_2_ monomer (Figure 4A,B). A typical branched polymer (Polymer-1) was obtained through the Michael addition reaction^30^ (the detailed experiment process is described in the Supporting Information). The graphs of weight-average molecular weight (*M*_w_), number-average molecular weight (*M*_n_), and polydispersity (*Đ*) were plotted against A reaction extent in Figure 4C–E.

**Figure 4:**
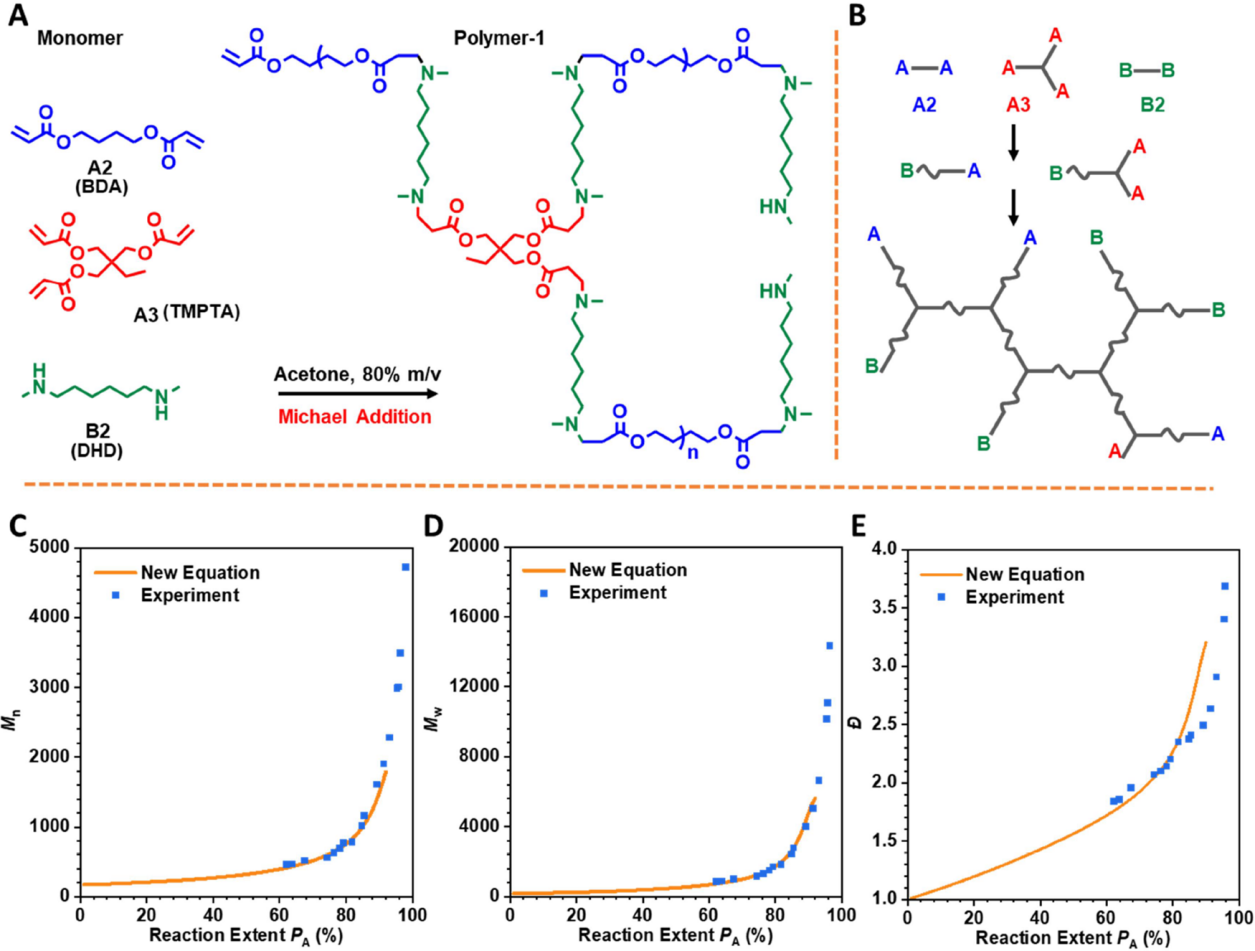
Experimental polymerization behavior of BDA + TMPTA + DHD. (A) Chemical structure of monomers and Polymer-1. (B) Schematic representation of the polymerization process of the BDA + TMPTA + DHD system. Comparison of the (C) weight-average molecular weight (*M*_w_), (D) number-average molecular weight (*M*_n_), and (E) polydispersity (*Đ*) obtained from the new equations, MC simulations, and real experiment. The initial molar ratio of BDA:TMPTA:DHD was set as 1:0.1:1.15. Experimental *M*_w_, *M*_n_, and *Đ* were measured by gel permeation chromatography (GPC) (Figure S3 and Table S1). The reaction extent of A was calculated from proton nuclear magnetic resonance (^1^H NMR) (Figures S4–S6).

The results show that in three distinct scenarios (*M*_w_, *M*_n_, *Đ*), the newly derived formulas demonstrate consistent evolution trends and levels as the real experiments. Deviation occurs only in the later reaction stages (*P*_A_ > 80%, approaching the gel point), where Flory’s theoretical framework becomes less applicable. Nevertheless, at all other stages, the results of the new formulas align well with experimental data, which is a highly positive outcome, indicating the great potential of these newly derived formulas regarding ${\overset{®}{X}}_{n}$ , ${\overset{®}{X}}_{w}$ and *Đ* in guiding both practical academic experiments and industrial production.

### A_2_ + A_3_ + B_2_ System—Branch Unit Distribution (BUD)

Next, the derivation of BUD was extended to the A_2_ + A_3_ + B_2_ system. Similarly, based on *w*′_*x*(AB)_, the proportion of branching unit A_3_ (i.e., the ratio of the total amount of A_3_ units in the molecules containing *x* A_2_ or A_3_ and *b* B_2_ to the total amount of A_2_, A_3_ and B_2_ in the system) was first calculated as follows:

wbx(AB)=∑z=1,3,5⋯∞nxx(x+b)Wz(x+b)Qxz/WAB(z(1−q))+n+b 29

The proportion of linear unit A_2_ – the ratio of the total amount of A_2_ units in the molecules containing *x* A_2_ or A_3_ and *b* B_2_ to the total amount of A_2_, A_3_ and B_2_ in the system – can also be obtained:

wlx(AB)=∑z=1,3,5⋯∞x−nxx(x+b)Wz(x+b)Qxz/WAB(z(1−q))+n+b 30

Therefore, combining eqs 29 and 30, the ratio of branched units A_3_ to linear units A_2_ in the molecule with *x* A_2_ or A_3_ units and *b* B_2_ units can be obtained:

$${\mathrm{BUD}}_{x}=\frac{wb_{x(\mathrm{AB})}}{wl_{x(\mathrm{AB})}}$$ 31

Similarly, the weight-average degree of polymerization ( $\overset{®}{x}$ ) can be obtained for a group of molecules with similar sizes.

x®x=∑xx+gap−1xwx(AB)′∑xx+gap−1wx(AB)′=∑xx+gap−1(x∑z=1,3,5⋯∞Wz(x+b)Qxz′/WAB(z(1−q))+n+b)∑xx+gap−1(∑z=1,3,5⋯∞Wz(x+b)Qxz′/WAB(z(1−q))+n+b) 32

Furthermore, by combining the *w*′*_*x*_* values of these molecules, we can calculate the average ratio of A_3_ monomers to A_2_ monomers in the polymer component with a weight-average degree of polymerization of $\overset{®}{x}$ :

$${\mathrm{BUD}}_{\overset{®}{x}}=\frac{\sum_{x}^{x+gap-1}w_{x(\mathrm{AB})}^{\prime }\frac{Wb_{x(\mathrm{AB})}}{Wl_{x}(\mathrm{AB})}}{\sum_{x}^{x+gap-1}w_{x(\mathrm{AB})}^{\prime }},\;x=1,gap+1,2gap+1,3gap+1,\cdot \cdot \cdot$$ 33

Similar to the A_2_ + A_3_ system, the plotted curves of BUD vs $\;\overset{®}{x}$ decrease as the reaction extent increases (Figure S7). Additionally, at the same weight-average degree of polymerization, the BUD is independent of the *gap* values.

Finally, the newly derived BUD formula was compared with that measured in real experiments. An equimolar A_2_ + A_3_ + B_2_ SGP system was carried out under the same monomer types, experimental conditions, and procedures as described before (Figure 4A). When polymerization finished, excessive DHD was added to the reaction to eliminate unreacted acrylate groups. After obtaining the product (Polymer-2, Figure 5A), different polymer components (P1–P8) of varying molecular weights were separated through elution fractionation (Figure 5B), and the BUD were calculated by ^1^H NMR (Figures S8, S9, Table S2, experimental and characterization methods were described in the Supporting Information).

**Figure 5:**
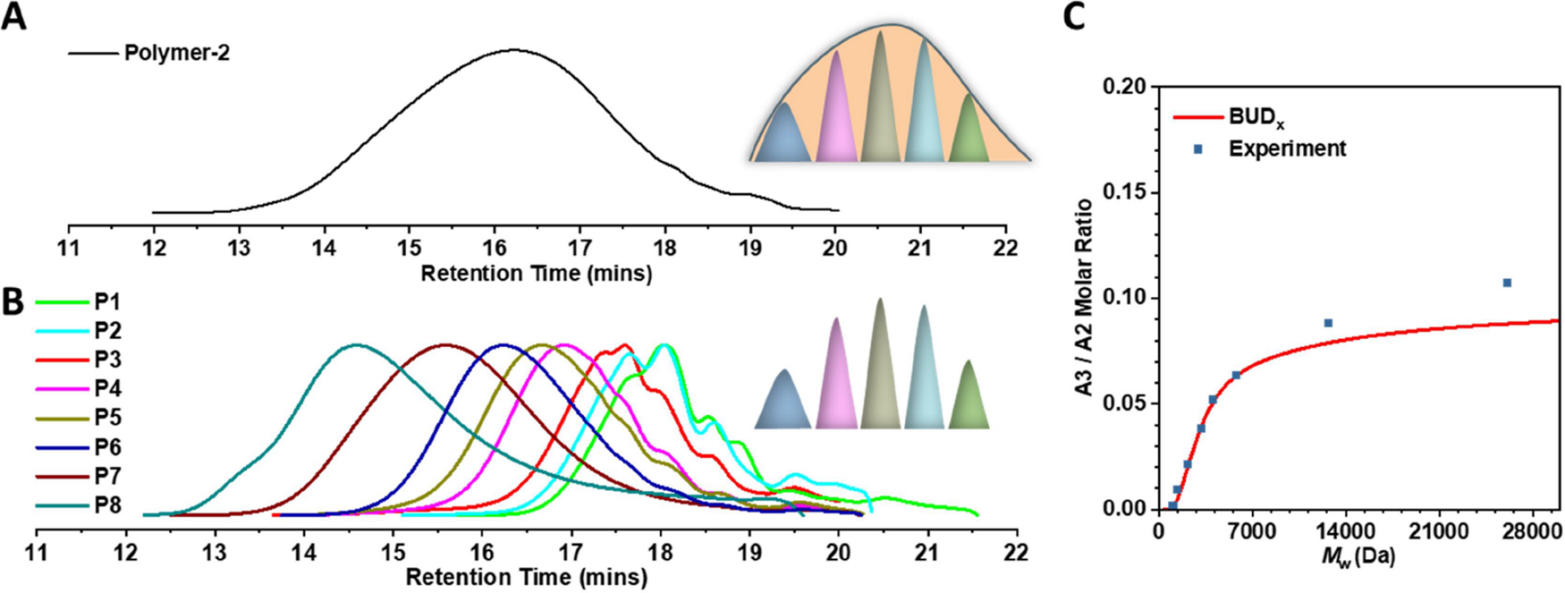
BUD in the BDA:TMPTA:DHD system. (A) GPC traces of original polymer (Polymer-2, before fractionation). (B) GPC traces of different molecular weight components in Polymer-2. (C) Comparison of BUD obtained from eqs 32 and 33 and the experiment. The initial molar ratio of BDA:TMPTA:DHD was set as 1:0.1:1.15. *M*_w_ was obtained from gel permeation chromatography (GPC) (Table S2). BUD was calculated from proton nuclear magnetic resonance (^1^H NMR) (Figures S5 and S6). The A reaction extent of experiment was around 95%.

Figure 5B and Table S2 show the GPC and ^1^H NMR characterization results for each individual component P1 to P8 fractionated from Polymer-2. These data clearly illustrated the parallel movements of the molecular weights of P1 to P8 from low to high, *M*_w_ = 1105 to 26,131 Da. Then the ratios of TMPTA units to BDA units on each polymer component (P1 to P8) were calculated here to evaluate the BUD. Through a comparison of the derived formula and experimental data, a consistent trend of BUD was obtained – the A_3_/ A_2_ molar ratio increases as the molecular weight of the components increases, and then gradually stabilizes (Figure 5C).

The BUD observed in the above study is merely a statistical average, but polymers are composed of various molecules, and not all molecules within the same molecular weight range share identical structures. To further uncover the molecular structural patterns behind this BUD phenomenon, we used MC simulation (see detailed simulation description in Supporting Information, Scheme S3). In this simulation, the connection information for each monomer in the polymer, that is, the structure of each molecule, was recorded. After reaching the set conversion rate, we extracted the structures of molecules within three different molecular weight regions for comparison (Figure 6). The total molecular weight (MW) of the molecules chosen within each region was set to be the same, approximately 20 kDa. As shown in Figure 6, within the low molecular weight region (MW ∼ 2000 Da), not all molecules contain branching units, with most molecules being linear. As the molecular weight increases, the content of linear molecules decreases, eventually forming complex branched structures. It is the first time that the phenomenon of BUD is theoretically validated, filling a blank field in the theory of branched polymers.

**Figure 6:**
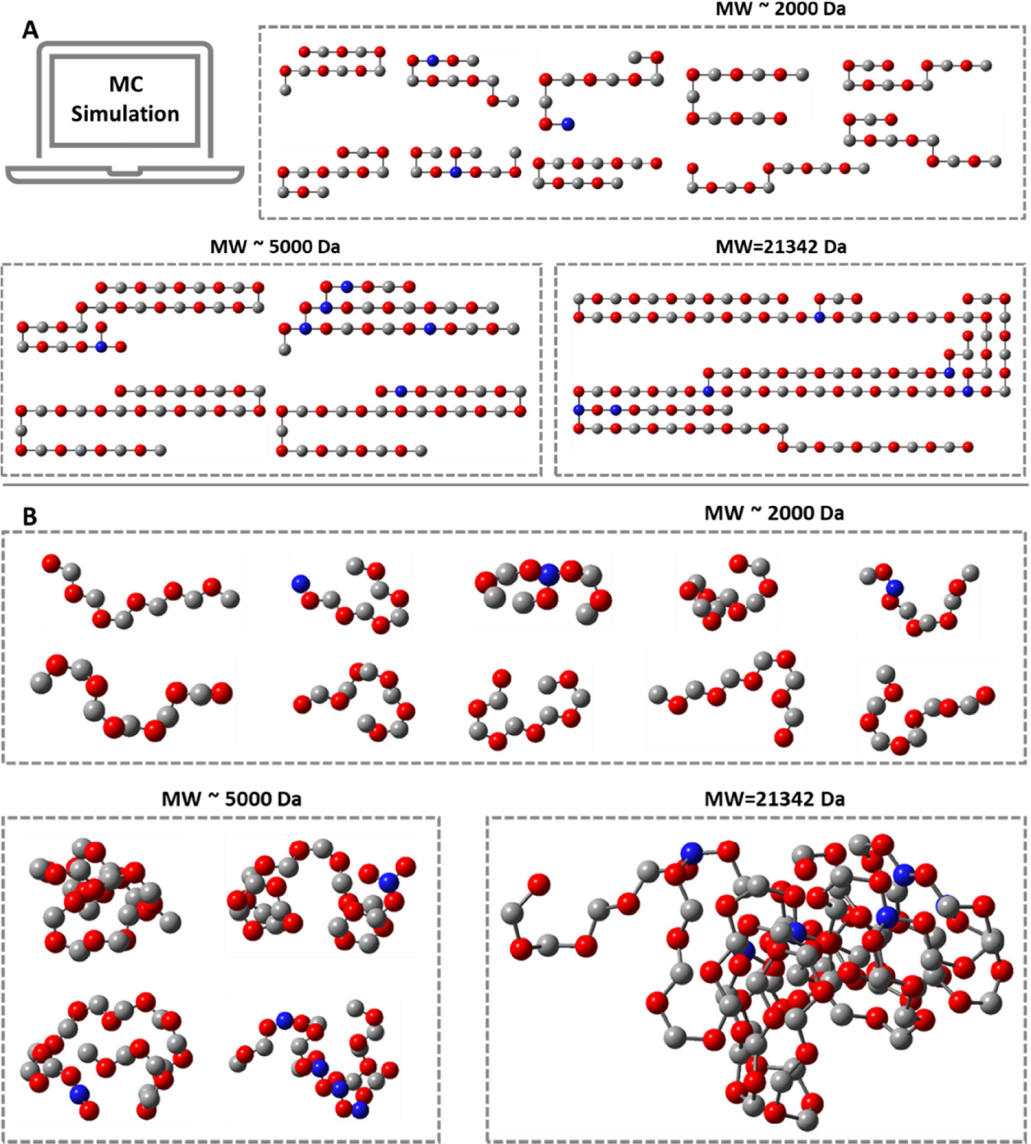
Structure of macromolecules with different MWs. (A) Flattened molecular structure. (B) Relaxed 3D structure. Molecule structures were obtained based on MC simulation results. The blue spheres represent TMPTA monomers, the red spheres represent DHD, and the gray spheres represent BDA monomers. The A reaction extent in the MC simulation was 95%.

### BUD in Future Polymerization Analysis

In this work, BUD was studied only in the A_2_ + A_3_ and A_2_ + A_3_ + B_2_ systems. In future research, it will be further extended to systems such as AB_2_ + AB,^18^ AB_g_ + B_f_,^19,20^ and AB_g_ + AB_h_ + B_f_,^21^ A_2_ + A_n_ + B_2_,^22–24^ as well as other copolymer systems. This will provide new insights into the study of branched and hyperbranched polymers. Additionally, BUD can be extended as a broader concept, which refers to the distribution of different monomers in various molecular weight fractions within copolymer systems. Given that polymers always appear as mixtures of components with different molecular weights, these distributions influence the overall properties of the polymer. Future work will explore these aspects from both theoretical and experimental perspectives.

## Conclusions

The unfinished theoretic work by Flory in the A_2_ + A_3_ and A_2_ + A_3_ + B_2_ polymerization systems is successfully extended. In this study, the two aspects untouched by Flory’s 1941 work have been developed: (1) Building upon the revised weight fraction distribution, analytical expressions for *X*_n_, *X*_w_, and *Đ* were further derived through Flory’s probability method. (2) The internal structure of polymers was considered. For the first time, an analytical derivation and verification of the distribution of branched units within branched polymers was achieved. Furthermore, the newly developed theoretical framework aligns well with experimental results, reinforcing its validity. This work is expected to contribute significantly to the advancement of step-growth polymerization theory.

## Supplementary Material



## Notes

Detailed experimental procedures; calculated weight fraction distributions; BUD, *X*_w_, *X*_n_, and Đ traces in A_2_ + A_3_ + B_2_ system; GPC trace measurements; tables of molecular weight and reaction extent results, and schemes of the Monte Carlo simulation process (PDF)
