# Supplementary material for: Probability Method of Molecular Size, Polydispersity, and Branch Unit Distribution Function for the Three-Dimensional Polymers: A Continuation of Flory’s Work in 1941
Source: Macromolecules. 2025 May 21;58(16):8934–42. doi: 10.1021/acs.macromol.5c00090 (PMC12392732; doi:10.1021/acs.macromol.5c00090)
Supplement: Supplementary file 1 [file ma5c00090_si_001.pdf]

Supporting Information

Probability Method of Molecular Size, Polydispersity, and Branch Unit Distribution Function for the Three-Dimensional Polymers: A Continuation of Flory's Work in 1941

Yinghao Li,† Jing Lyu,*,† and Wenxin Wang*,†

†*Charles Institute of Dermatology, School of Medicine, University College Dublin, Dublin 4, Ireland, D04V1W8*

1. Materials

1,4-butanediol diacrylate (BDA), trimethylolpropane triacrylate (TMPTA) and N,N′-Dimethyl-1,6-hexanediamine (DHD) were purchased from Sigma-Aldrich. Lithium bromide (LiBr) for GPC measurements was purchased from Sigma-Aldrich. Acetone, dimethylformamide (DMF) and hexane were purchased from Fisher Scientific. Deuterated chloroform ($CDCl_3$) was purchased from Sigma-Aldrich.

2. Polymer Synthesis

Polymer-1 and -2 were synthesized through a Michael addition reaction. Specifically, 1,4-butanediol diacrylate (3.96 g), trimethylolpropane triacrylate (0.592 g) and N,N′-Dimethyl-1,6-hexanediamine (3.32 g) were dissolved into 80% weight/volume (w/v) with acetone. Then the reactions were carried out in the ice bath. Agilent 1260 Infinite gel permeation chromatography (GPC) and nuclear magnetic resonance (NMR) were used to monitor the reaction. The reaction was stopped by adding 3.32 g DHD when M_w was approaching 10 kDa at room temperature for 24 h. After that, Polymers were precipitated into hexane for purification and dried under vacuum before being stored at −20 °C.

3. Polymer Fractionation

After achieving Polymer-2, it was fractionated to obtain different components of a range of molecular weights (P1 to P8). The fractionation procedure is as follows: Polymer-2 was dissolved in acetone of a concentration of 100 mg/ml, then the solution was slowly added into the mixed solvent of acetone and hexane (v/v=1/9) under gentle agitation at room temperature. The solvent in the supernatant solution was removed by

rotary evaporator and the product was collected as component P1. Then the precipitate was redissolved in acetone and precipitated into another mixed solvents with higher acetone extent (acetone/hexane =2/8) to generate component P2. By repeating the step-by-step precipitation process, Components P1 to P8 were obtained.

4. Polymer Characterization

An Agilent 1260 Infinite gel permeation chromatography (GPC) equipped with a triple detector (a refractive index detector (RI), a viscometer detector (VS DP) and a dual light scattering detector (LS 15° and LS 90°)), was used to monitor the change of functional groups conversion rate, the weight-average molecular weight (M_w), number-average molecular weight (M_n), and polydispersity ($Đ$). For GPC measurement, 100 µl of the reaction mixture was taken and diluted in 1ml DMF, and filtered through a 0.45 µm filter. DMF with 0.1% LiBr was utilized to elute the GPC columns (Polar Gel-M, 7.5×300 mm, two in series) at a flow rate of 1ml/min at 60 ^{o}C. Linear poly(methyl methacrylate) (PMMA) standards were used for the calibration of the GPC columns. Meanwhile, the conversion rates and chemical compositions were monitored with ^{1}H NMR on a 400 MHz Varian Inova spectrometer. The samples were reported in parts per million (ppm) relative to the solvent $CDCl_3$ (7.23 ppm) or internal control (tetramethylsilane 0.00 ppm).

5. Monte Carlo Simulation

The simulation uses a coarse-grained probabilistic model that does not take the specific molecular structures into account. In short, all reactants are randomly placed in a virtual reaction space without specific coordinates or locations. The size, dimensions, and

chemical structure details of the species in the space are ignored. Additionally, all components in the system are assumed to be fully accessible to each other. This ideal system strictly follows Flory's ideal system setup, meaning no intramolecular cyclization reactions occur, and all functional group reactivities are considered equal. All fundamental reactions occur between two randomly selected reactants in the system. In both models, information about macromolecules and monomers is recorded using a series of continuous numbers, which allows for distinguishing between different macromolecules and monomers. For the A_2+A_3 polymerization system, the program's operation flow is shown in **Scheme S1**. In simple terms, monomers undergo polymerization, and the reaction stops once a set degree of reaction is reached. The average degree of polymerization and polydispersity at different reaction extents are then calculated and recorded.

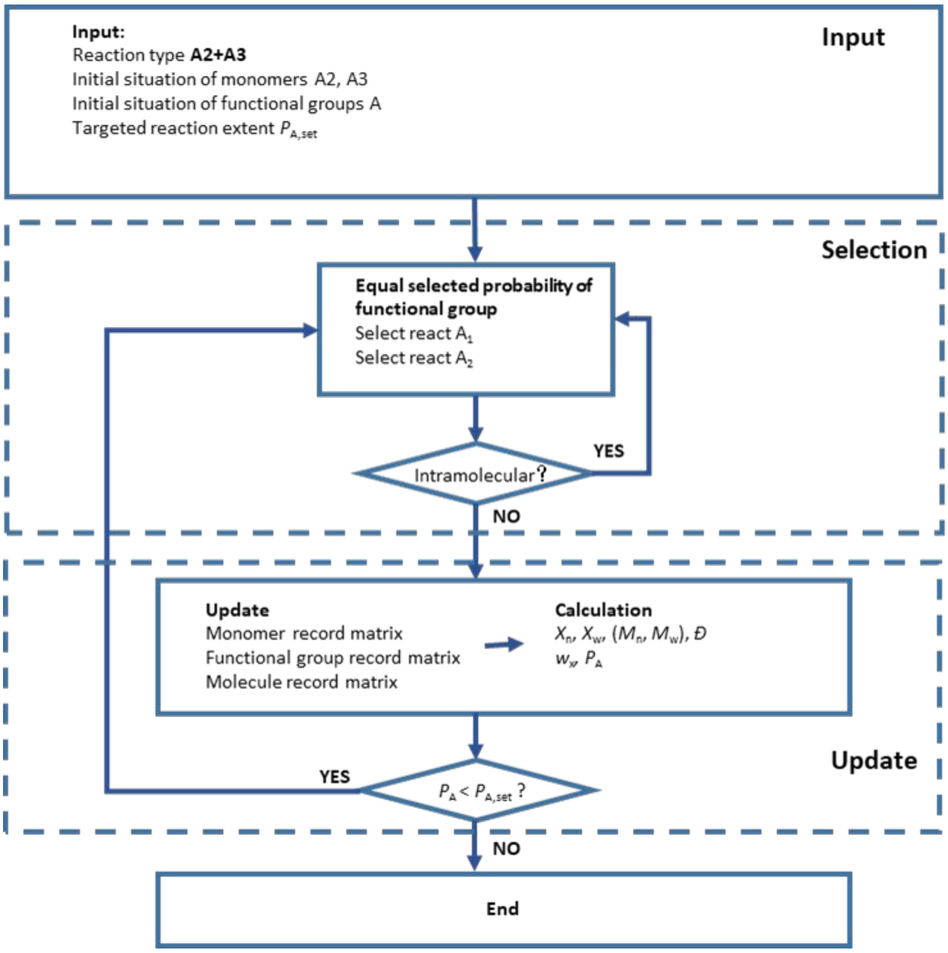

Scheme S1. Summary of main steps in the kinetic Monte Carlo model of A_2+A_3 reaction.

For the Branch Unit Distribution (BUD) simulation study of the A_2+A_3 system, the system setup and polymerization approach are the same as described in **Scheme S1**. It also follows the assumptions of no intramolecular cyclization reactions and equal probability for all functional group reactivities. The difference lies in the analysis performed after the polymerization reaction reaches the set degree of conversion and stops. At this point, further analysis is carried out on all macromolecules and remaining monomers. Based on a predefined 'gap' value, these macromolecules and monomers are

grouped according to their degree of polymerization. For each group, the number of A_2 and A_3 monomers and their weight-average degree of polymerization are calculated. Finally, the BUD results are obtained. The program's operation flow is shown in **Scheme S2**.

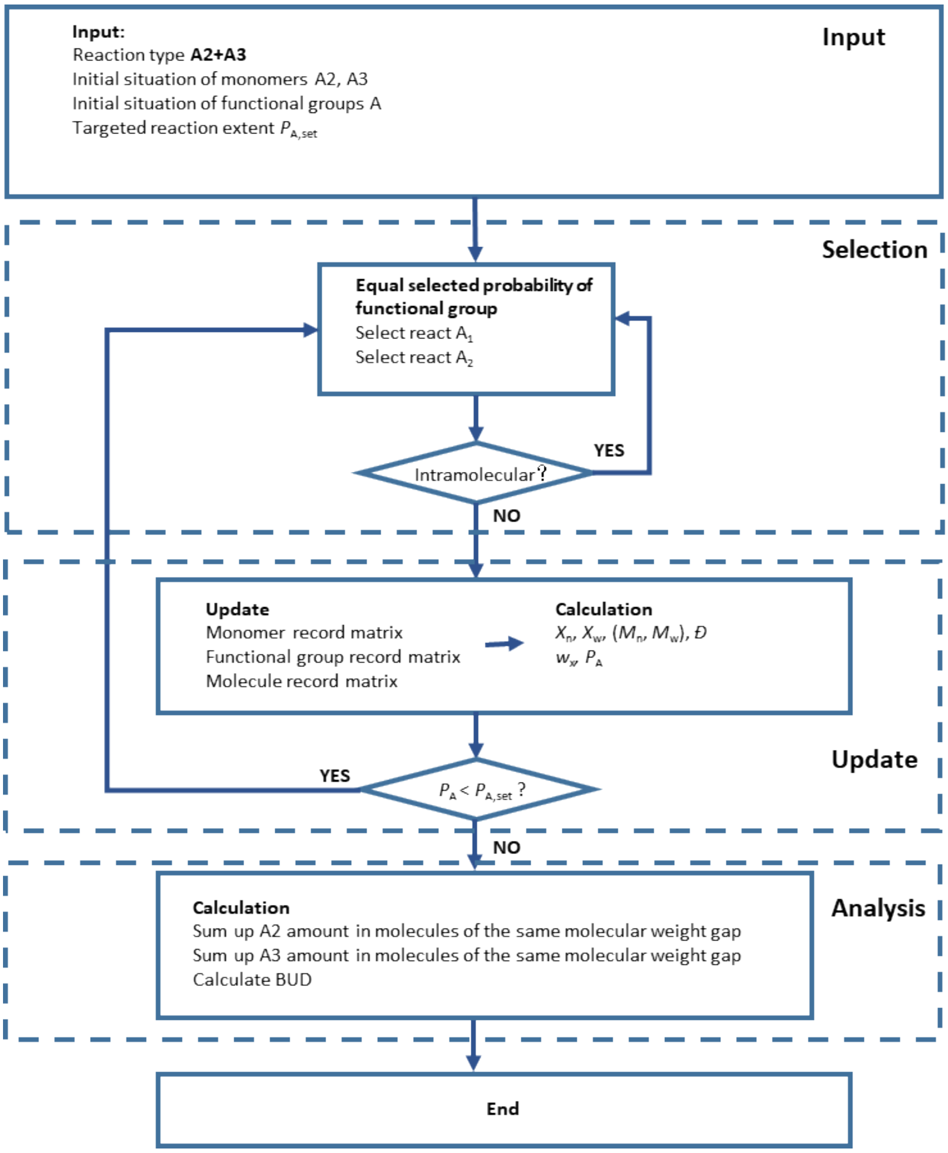

Scheme S2. Summary of main steps in the kinetic Monte Carlo model of BUD in A_2+ A_3 reaction.

For the molecular structure simulation study of the A_2+A_3+B_2 system, the system setup and polymerization method are similar to those described in Scheme S2. It also follows the assumptions of no intramolecular cyclization reactions and equal reactivity probability for all functional groups. The analysis is conducted after the polymerization reaction reaches a set conversion rate and stops. At this point, molecules with a specified molecular weight are randomly selected, and information is extracted, including the identification numbers of all monomers comprising the molecule and bond connection details (i.e., which two monomers are connected). Subsequently, a spatial coordinate system is established, placing the first monomer of the molecule at the center, and positioning the next monomer according to bond connection information. The positioning of monomers is randomly selected but follows these principles: monomers cannot overlap, bonds cannot cross or break, and the molecular size is fixed.

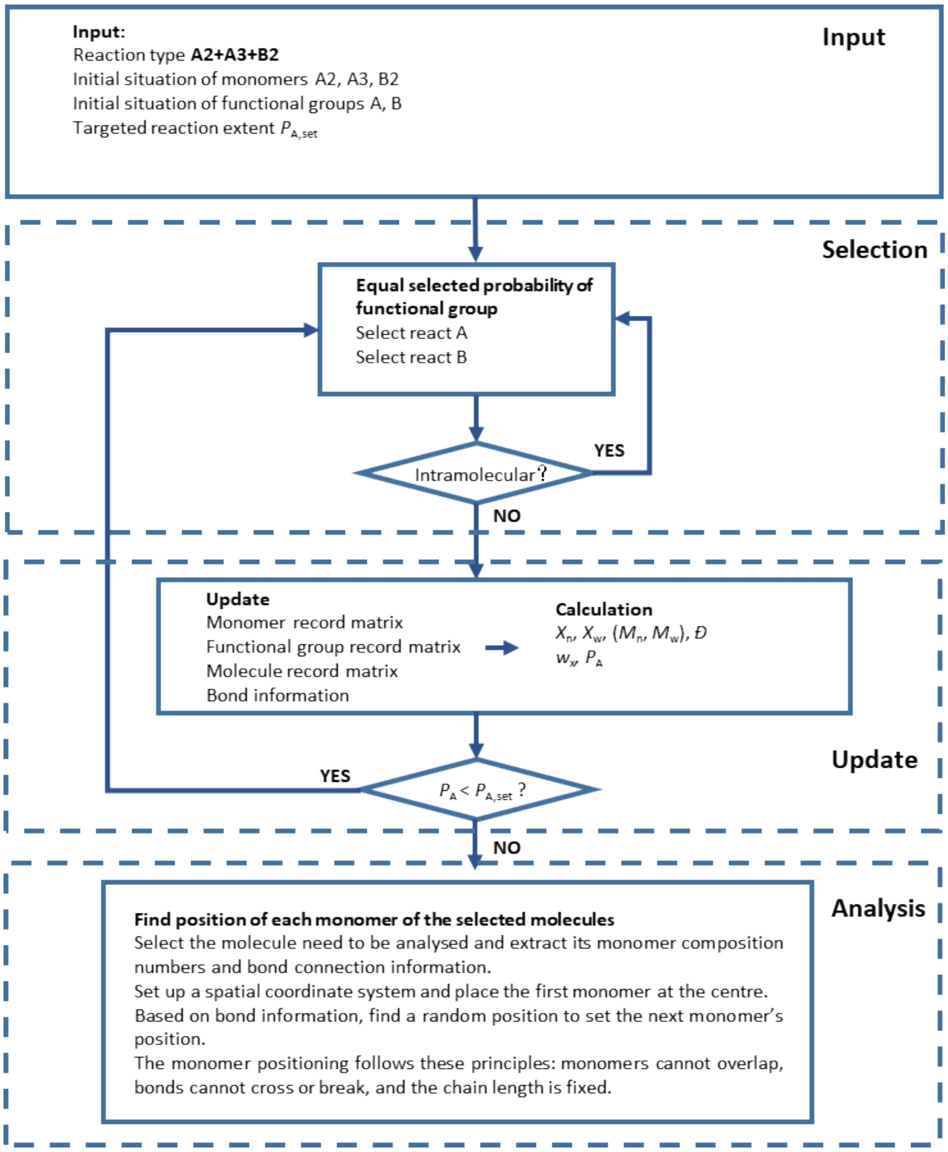

Scheme S3. Summary of main steps in the Monte Carlo model in A_2+A_3+B_2 reaction.

6. Supporting data

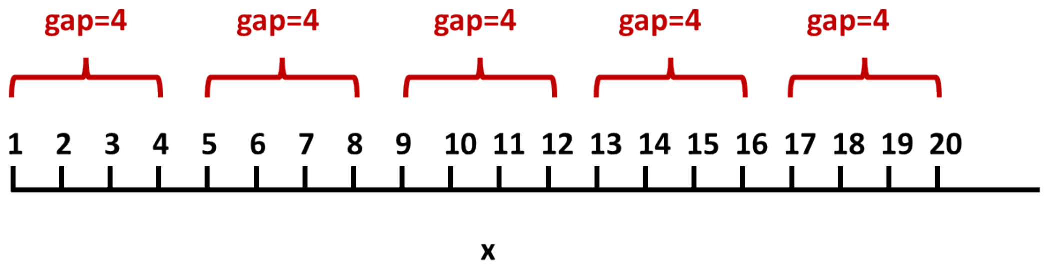

Scheme S4. Example of how to use gap to split polymer components with different molecular size into different groups.

Flory

$$\begin{cases} Q_{xz} = \sum[(1-q)q^{y_1-1}(1-q)q^{y_2-1}\cdots(1-q)q^{y_z-1}] \\ y_1 + y_2 + \cdots y_z = x \\ Q'_{xz} = q^{x-z}(1-q)^z \frac{(x-1)!}{(z-1)!(x-z)!} \end{cases}$$

This work

$$\begin{cases} Q_{xz} = \sum[(1-q)q^{y_1-1}(1-q)q^{y_2-1}\cdots(1-q)q^{y_z-1}] \\ y_1 + y_2 + \cdots y_z = x - n \\ Q'_{xz} = q^{(x-n)-z}(1-q)^z \frac{((x-n)-1)!}{(z-1)!((x-n)-z)!} \end{cases}$$

Flory

$$\left\{ w_x = \sum_{z=1,3,5\cdots}^{\infty} \frac{W_z x Q_{xz}}{\left(z/_{(1-q)}\right)} \right.$$

This work

$$\begin{cases} w'_x = \sum_{z=1,3,5\cdots}^{\infty} \frac{W_z x Q'_{xz}}{\left(z/_{(1-q)}\right)+n} \\ wb_x = \frac{n}{x} w'_x = \sum_{z=1,3,5\cdots}^{\infty} \frac{n}{x} \frac{W_z x Q'_{xz}}{\left(z/_{(1-q)}\right)+n} \\ wl_x = \sum_{z=1,3,5\cdots}^{\infty} \frac{x-n}{x} \frac{W_z x Q'_{xz}}{\left(z/_{(1-q)}\right)+n} \\ BUD_x = \frac{Wb_x}{Wl_x} \end{cases}$$

Scheme S5. Illustration of the transformation of Flory's formula to the formula in this work.

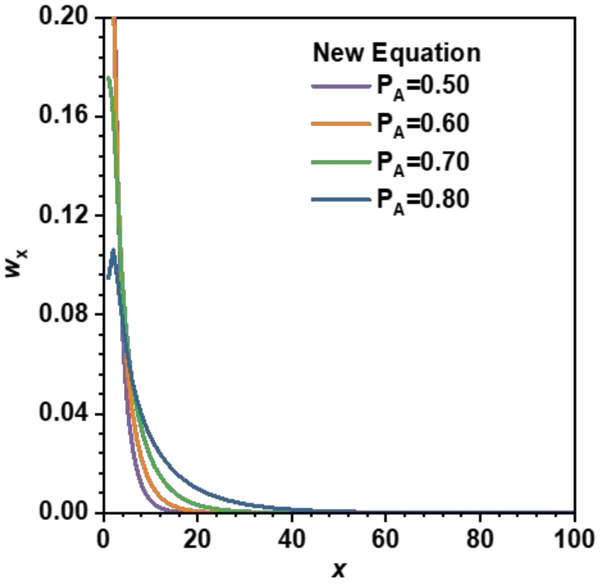

Figure S1. Weight fraction distributions of species in A_2+A_3+B_2 system under different reaction extent ($P_A = 0.80$, 0.70, 0.60, and 0.50). The initial molar ratio of A_2:A_3:B_2 was set as 1:0.1:1.15.

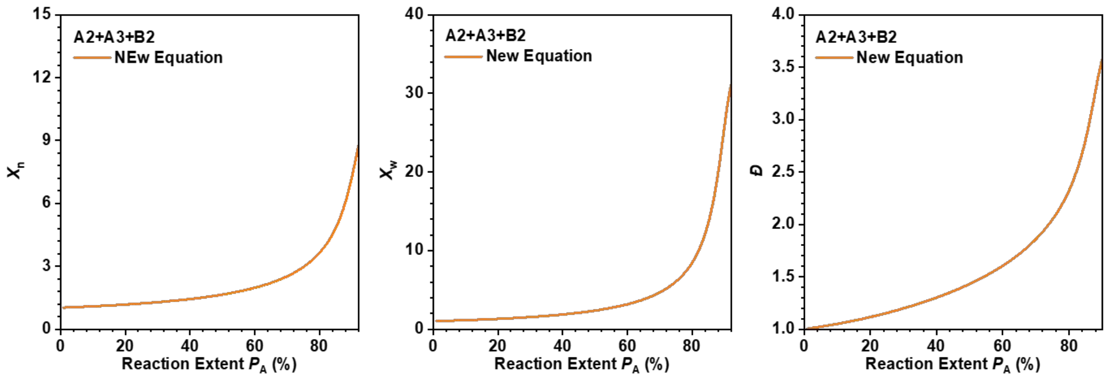

Figure S2. The polymerization behavior of A_2+A_3+B_2 system. Evolution of (A) weight-average degree of polymerization (X_w), (B) number-average degree of polymerization (X_n) and (C) polydispersity (*Đ*) according to the new equations proposed in this study (Equations (24), (25), and (26)), and Monte Carlo simulation. The initial molar ratio of A_2:A_3:B_2 was set as 1:0.1:1.15.

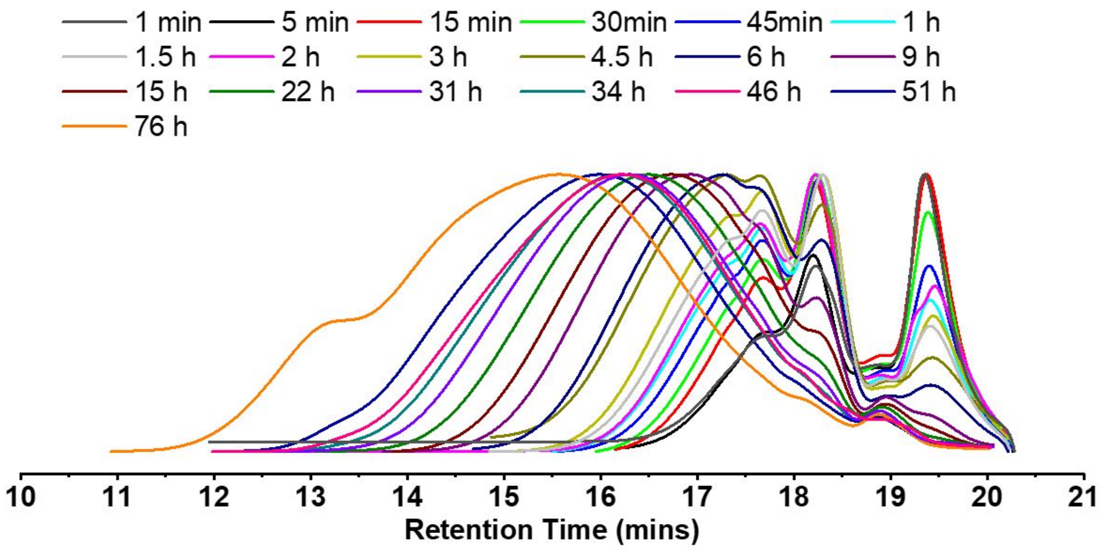

Figure S3. GPC traces of the Polymer-1 propagation.

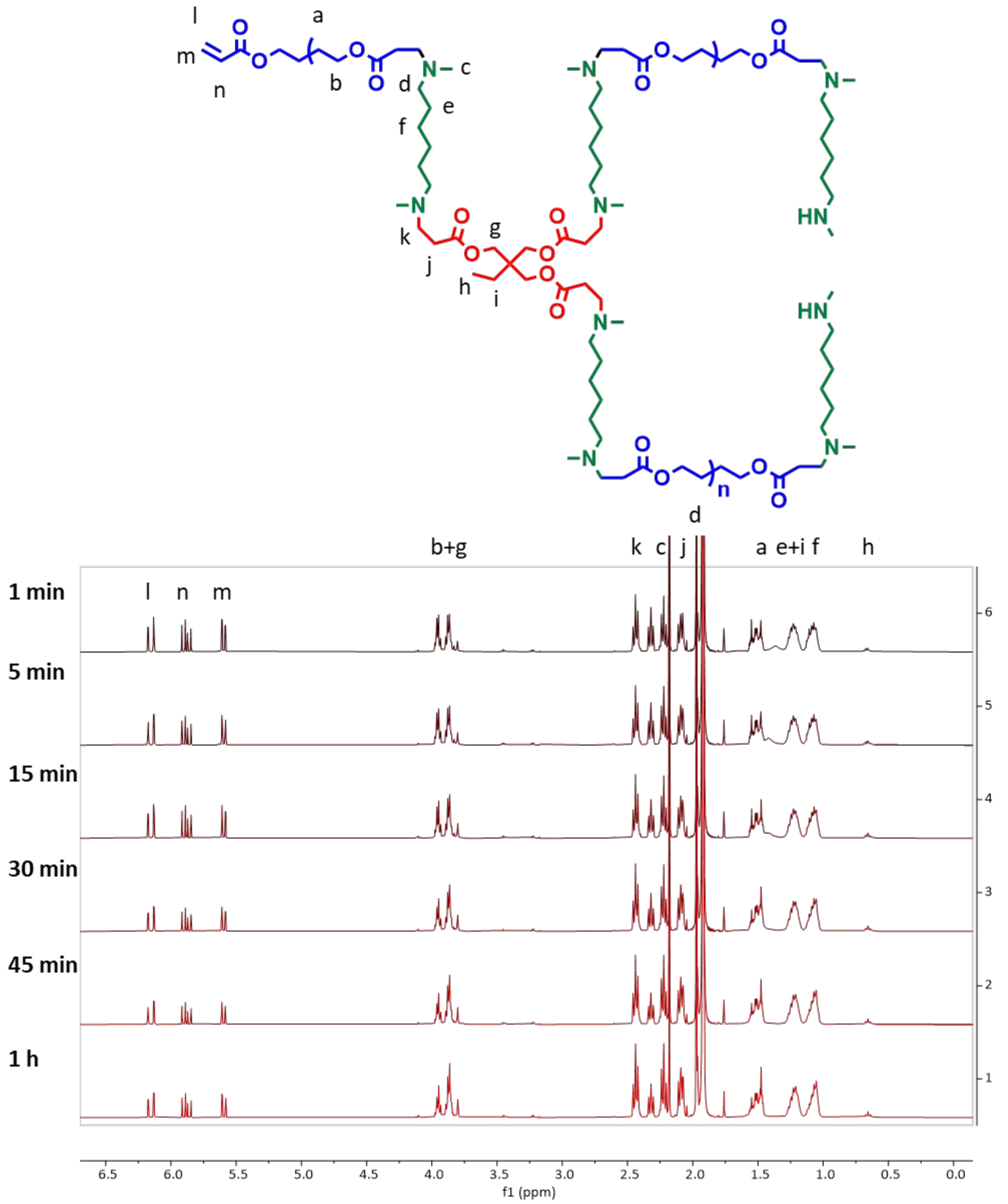

Figure S4. ^{1}H NMR spectra of the Polymer-1 propagation from 1 minute to 1 hour.

Acrylate (A) reaction extent = $[1-(I_m \times 2) / [I_a + I_h \times 3/2] \times 100\%$

where I_m, I_a and I_h stand for the integral intensity of peak m, a and h in ^{1}H NMR spectra.

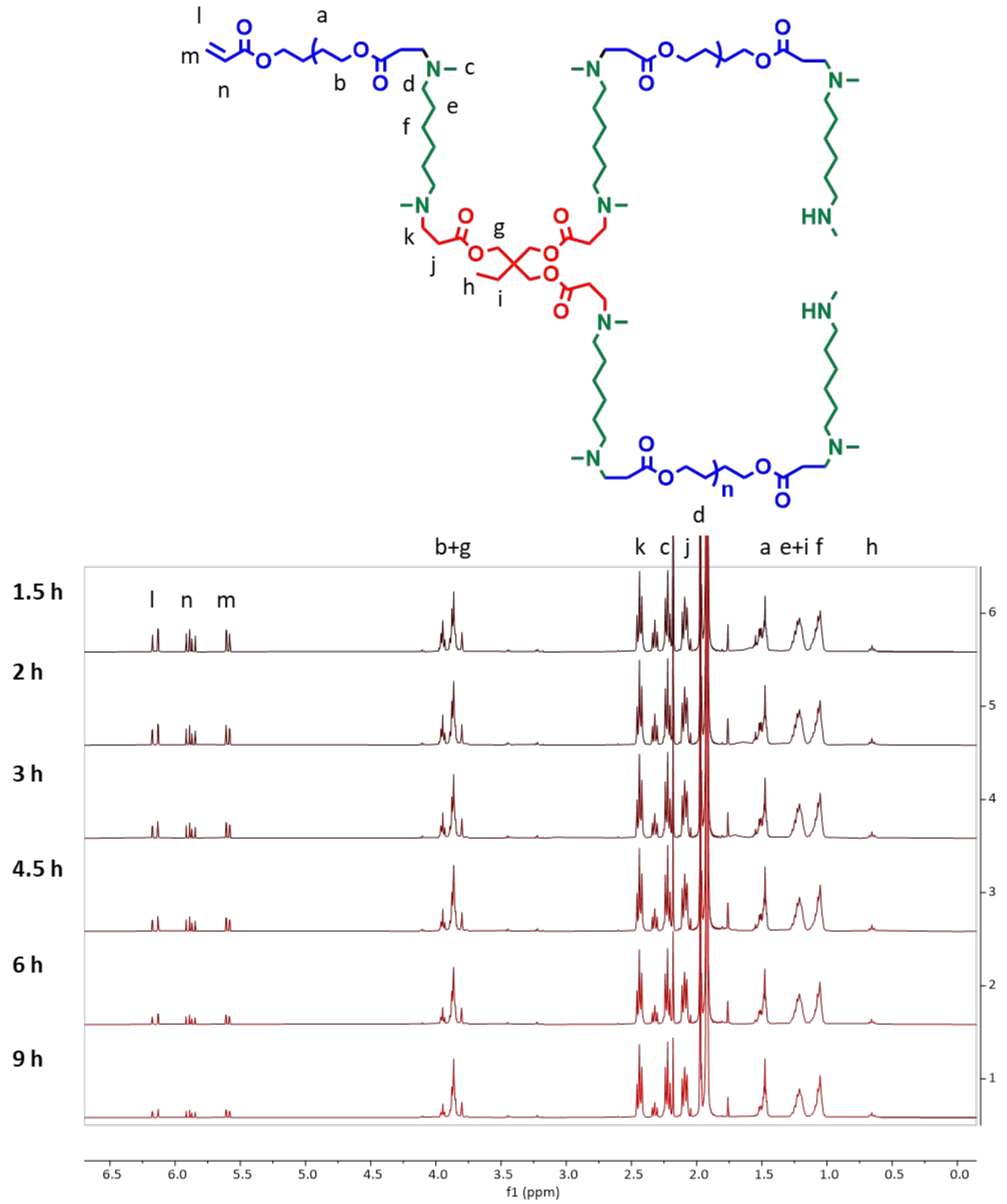

Figure S5. ^{1}H NMR spectra of the Polymer-1 propagation from 1.5 hours to 9 hours.

Acrylate (A) reaction extent = $[1-(I_m \times 2) / [I_a + I_h \times 3/2] \times 100\%$

where I_m, I_a and I_h stand for the integral intensity of peak m, a and h in ^{1}H NMR spectra.

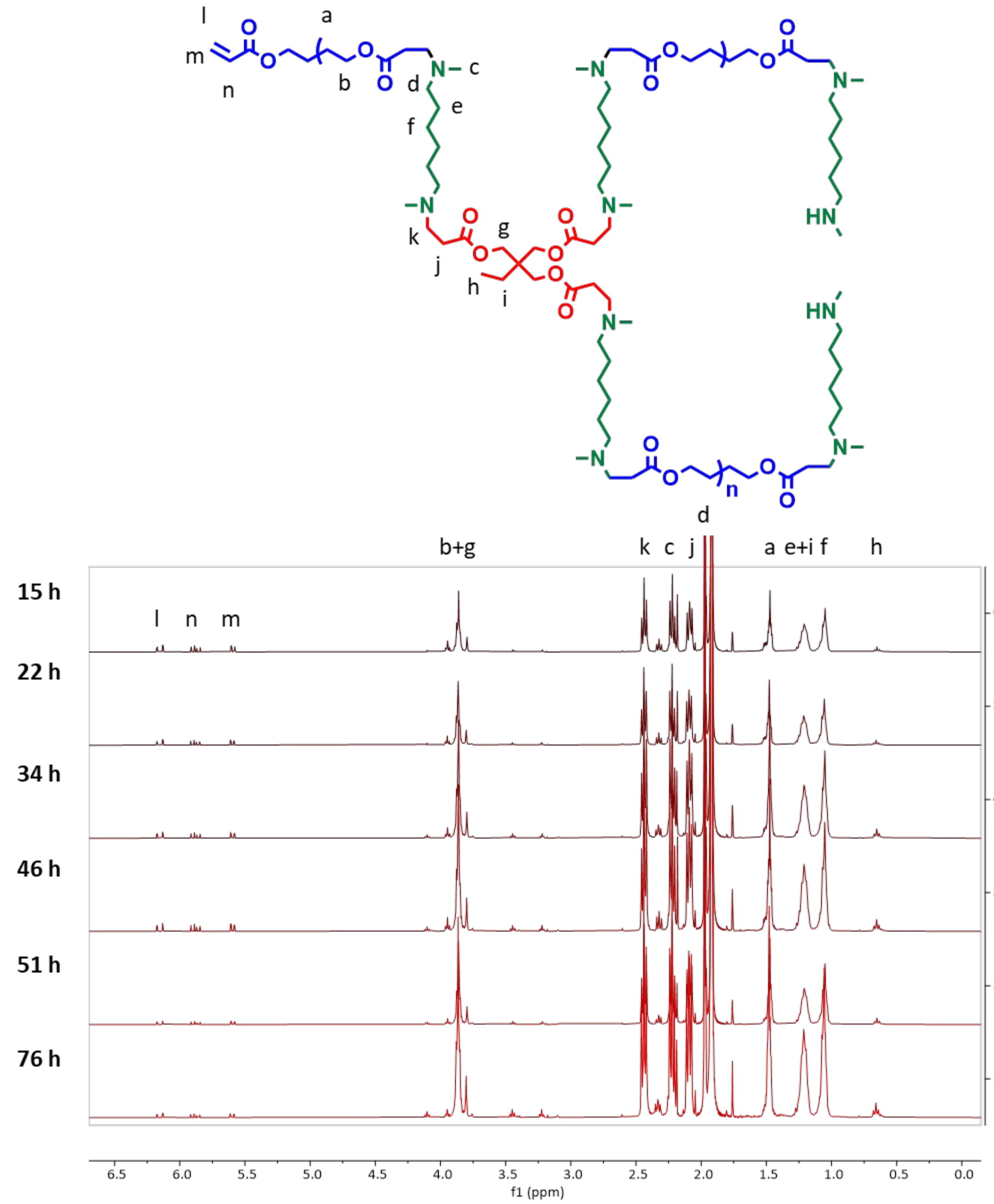

Figure S6. ^{1}H NMR spectra of the Polymer-1 propagation from 15 hours to 76 hours.

Acrylate (A) reaction extent = $[1-(I_m \times 2) / [I_a + I_h \times 3/2] \times 100\%$

where I_m, I_a and I_h stand for the integral intensity of peak m, a and h in ^{1}H NMR spectra.

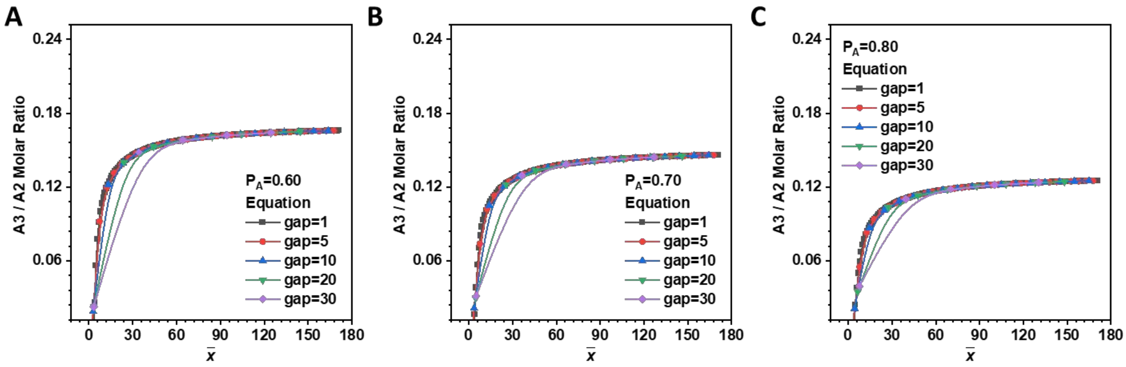

Figure S7. The BUD (molar ratio of A_3 to A_2 in A_2+A_3+B_2 system, obtained from Equation (35) and (36), at (A) $P_A = 0.6$, (B) $P_A = 0.7$, (C) $P_A = 0.8$. The initial molar ratio of A_2:A_3:B_2 was set as 1:0.1:1.15. Each Monte Carlo simulation under different conditions was repeated 10 times, and the average values were plotted.

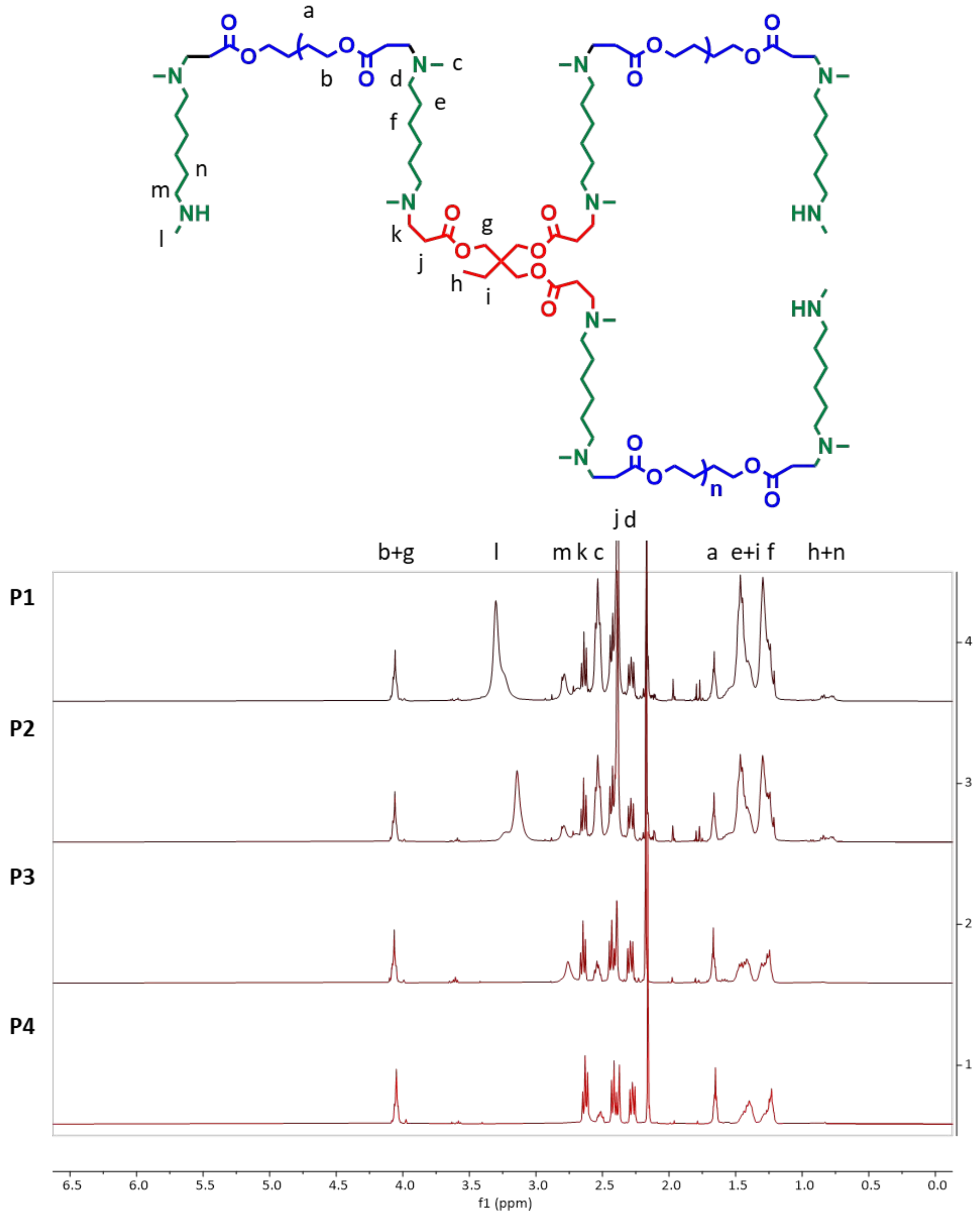

Figure S8. ^{1}H NMR spectra of the fractionation products P1 to P4 of Polymer-2.

BUD = Molar ratio calculation of [TMPTA] / [BDA] = $[(I_{b+g} - I_a) / 3\times2] / [I_a]$

where I_{b+g}, and I_a stand for the integral intensity of peak b+g and a in ^{1}H NMR spectra.

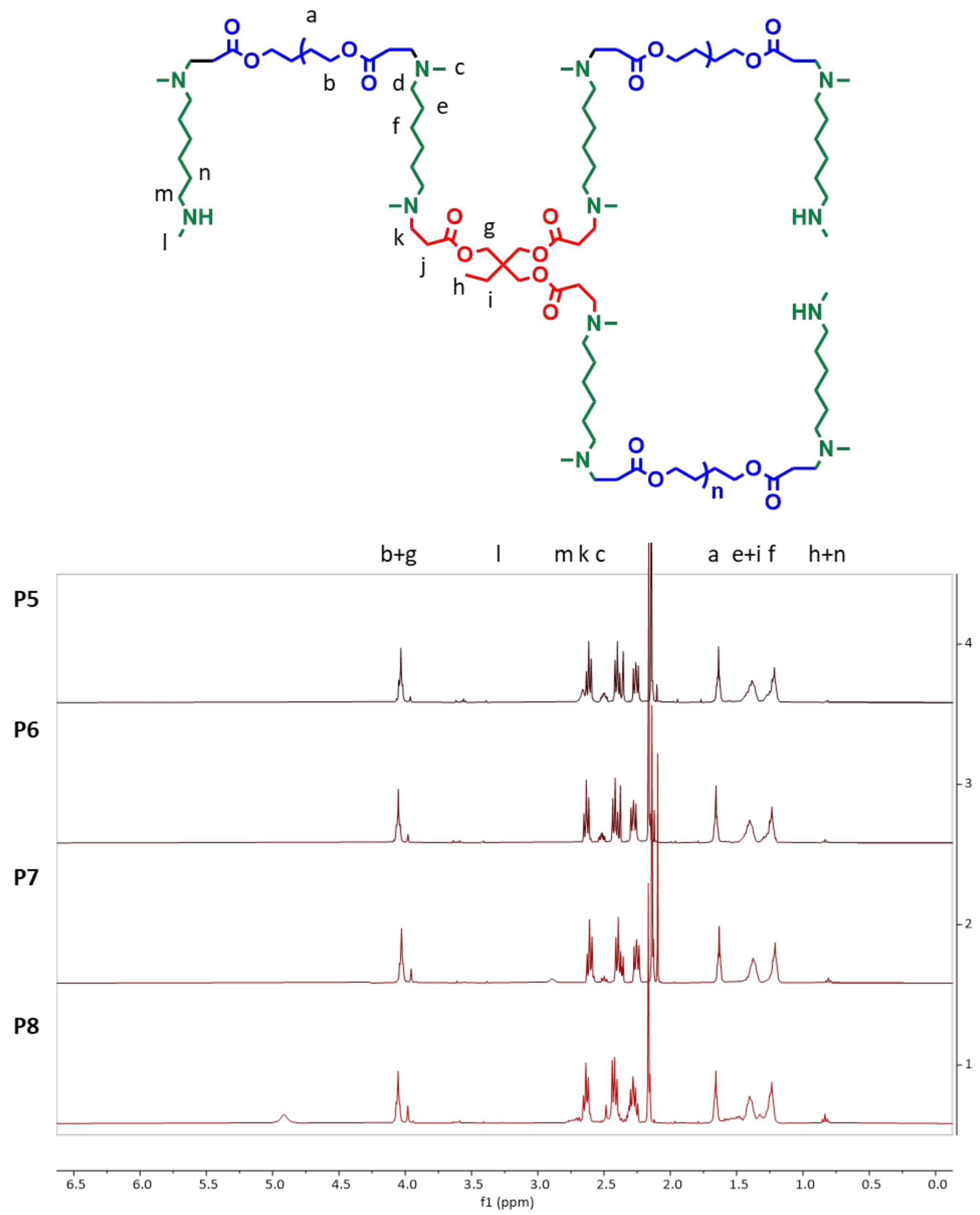

Figure S9. ^{1}H NMR spectra of the fractionation products P5 to P8 of Polymer-2.

BUD = Molar ratio calculation of [TMPTA] / [BDA] = $[(I_{b+g} - I_a) / 3\times2] / [I_a]$

where I_{b+g}, and I_a stand for the integral intensity of peak b+g and a in ^{1}H NMR spectra.

Table S1. GPC and NMR results of Polymer-1 during the polymerization process

	$M_{n,GPC}$ (Da)[a]	$M_{w,GPC}$ (Da)[a]	$Đ_{GPC}$[a]	Acrylate (A) reaction extent (%)[b]
1 min	455	837	1.84	62.23
5 min	465	862	1.85	63.90
15 min	515	1006	1.95	67.57
30 min	555	1148	2.07	74.44
45 min	629	1321	2.10	76.44
1 h	692	1480	2.14	78.26
1.5 h	768	1689	2.20	79.01
3 h	780	1831	2.35	81.82
4.5 h	1018	2418	2.38	84.84
6 h	1157	2785	2.41	85.62
9 h	1603	3996	2.49	89.26
15 h	1901	5008	2.63	91.40
22 h	2278	6621	2.91	93.20
34 h	2987	10158	3.40	95.46
51 h	3492	14331	4.10	96.48
76 h	4722	33542	7.10	97.98

[a] Determined by GPC RI detector. [b] Calculated from ^{1}H NMR spectra.

Table S2. Characterization results of Polymer-2 and fractionation products

	$M_{n,GPC}$ (Da)[a]	$M_{w,GPC}$ (Da)[a]	$Đ_{GPC}$[a]	BUD[b]
Polymer-2	3004	11073	3.69	-
P1	498	1105	2.22	0.001333
P2	673	1414	2.10	0.009333
P3	1038	2194	2.11	0.021333
P4	1709	3221	1.88	0.038
P5	1802	4106	2.28	0.052
P6	2695	5830	2.16	0.063333
P7	4084	12745	3.12	0.088
P8	4707	26131	5.55	0.107333

[a] Determined by GPC RI detector. [b] Calculated from ^{1}H NMR spectra.
